# Supplementary figures and images for: Extended-spectrum beta-lactamase-producing Enterobacterales in human health: Experience from the tricycle project, Ghana
Source: PLoS One. 2024 Nov 11;19(11):e0310058. doi: 10.1371/journal.pone.0310058 (PMC11554194; doi:10.1371/journal.pone.0310058)

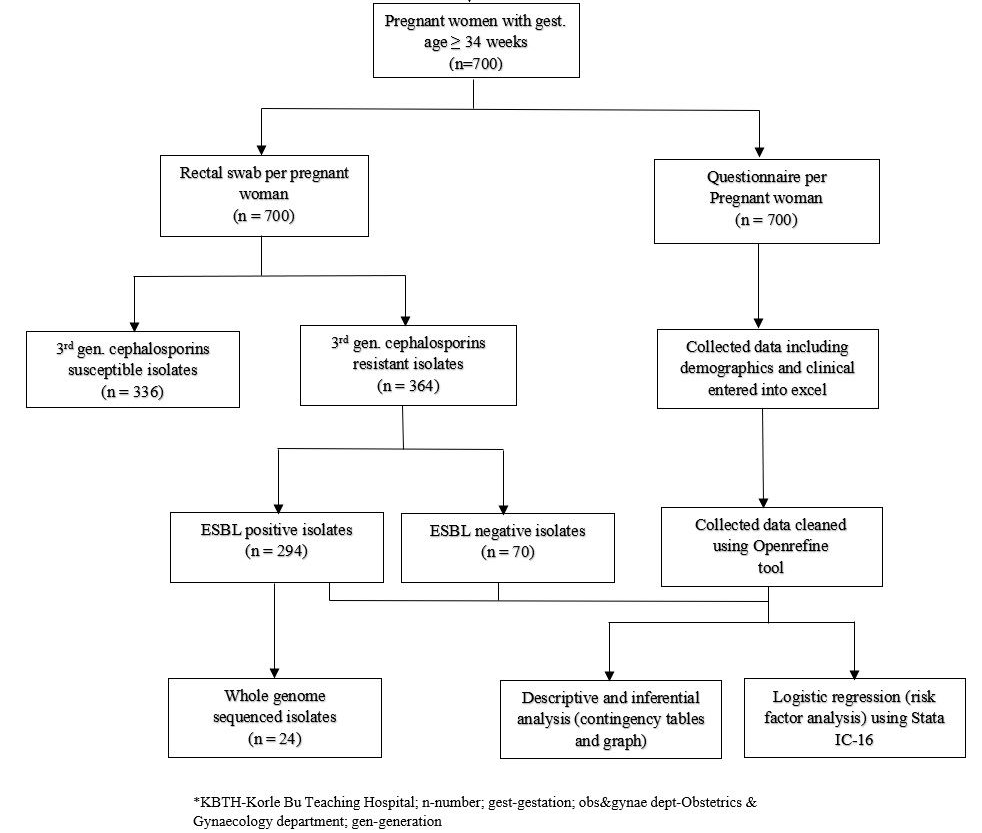

Supplement: S1 Fig — KBTH-Korle Bu Teaching Hospital; n-number; gest-gestation; obs&gynae dept-Obstetrics & Gynaecology department; gen-generation. (TIF) [file pone.0310058.s001.tif]
