# Supplementary material for: Extended-spectrum beta-lactamase-producing Enterobacterales in human health: Experience from the tricycle project, Ghana
Source: PLoS One. 2024 Nov 11;19(11):e0310058. doi: 10.1371/journal.pone.0310058 (PMC11554194; doi:10.1371/journal.pone.0310058)
Supplement: S1 Table — SD-Standard deviation; n-number, min- minimum, max- maximum. (PDF) [file pone.0310058.s002.pdf]

S2 Table Distribution of participants social demographics and clinical characteristics

Variable	Total number (n = 700)	Percentage (%)
Mean age in years (±SD)	31.54 (±5.50)	(min: 16 – max: 42)
**Age range**		
Adolescents	7	1
Adults	693	99
**Educational level**		
None	45	6.53
Primary	280	40.00
Secondary	206	29.33
Tertiary	169	24.14
**Married status**		
No	188	26.86
Yes	512	73.14
**Employment status**		
No	521	74.43
Yes	179	25.57
**Toilet facility at home**		
No	133	19.00
Yes	567	81.00
**Hand washing before eating**		
No	12	1.71
Yes	688	98.29
**Hand washing after defecation**		
No	5	0.71
Yes	695	99.29
**Hospitalization in the past year**		
No	551	78.71
Yes	149	21.29
**Antibiotic use in the past year**		
No	647	92.43
Yes	53	7.57
**Surgical procedure in the past year**		
No	676	96.57
Yes	24	3.43
**RVD status**		
Negative	682	97.43
Positive	13	1.86
Unknown	5	0.71
**ESBL phenotype**		
Negative	406	58.00

Positive	294	42.00

*SD-Standard deviation; n-number, min- minimum, max- maximum
