# Supplementary material for: Extended-spectrum beta-lactamase-producing Enterobacterales in human health: Experience from the tricycle project, Ghana
Source: PLoS One. 2024 Nov 11;19(11):e0310058. doi: 10.1371/journal.pone.0310058 (PMC11554194; doi:10.1371/journal.pone.0310058)
Supplement: Attachment — Submitted filename: scan0109.pdf [file pone.0310058.s003.pdf]

UNIVERSITY OF GHANA
MEDICAL SCHOOL
DEPARTMENT OF MEDICAL MICROBIOLOGY

INTEGRI PROCEDAMUS

Ref. No:....................................

20th May 2024

RE: PONE-D-24-17702
Extended-spectrum beta-lactamase producing Enterobacterales in human health: experience from the tricycle project, Ghana

Thank you for your feedback and thorough checks.

We indeed received ethical approval from the IRB of the Korle-Bu Teaching Hospital (***KBTH-STC/IRB/000168/2021***), before commencing the study. A copy of this approval is enclosed and the text in the current submission has been updated to reflect the change.

We are sorry for uploading an approval letter which was later received from the College of Health Sciences (CHS). The ethical approval from the CHS was given to my student (Paul Kwao), primarily for the Whole Genome Sequencing bit of the study.

Thank you.

Yours faithfully,

Japheth A. Opintan

P. O. BOX GP4236, Accra Ghana | **Telephone:** +233 (0) 302 666987 / 666988 | **Fax:** +233 (0) 302 336062
Email: microbiologyugms@ug.edu.gh | www.ugms.ug.edu.gh

UG
