# Supplementary material for: Extended-spectrum beta-lactamase-producing Enterobacterales in human health: Experience from the tricycle project, Ghana
Source: PLoS One. 2024 Nov 11;19(11):e0310058. doi: 10.1371/journal.pone.0310058 (PMC11554194; doi:10.1371/journal.pone.0310058)
Supplement: Attachment — Submitted filename: Response_to_Reviewers.docx [file pone.0310058.s004.docx]

PONE-D-24-21155 Extended-spectrum beta-lactamase producing Enterobacterals in human health: experience from the tricycle project, Ghana

| **Comments** | **Response/Revisions** | **Reference** |
| --- | --- | --- |
| Reviewer #1 | | |
| Method Lines 1-3. Information obtained from Korle Bu Teaching Hospital only is not reflective of the total population of Ghana, hence there might be a selection bias | We are grateful for the insightful comments.  Indeed, we agree with the reviewer that Information obtained from Korle Bu Teaching Hospital only is not reflective of the total population of Ghana.  This has been duly acknowledged as a limitation, to read,  ‘Our study is not without limitations. First, the study was limited to only one tertiary hospital in Ghana, and the data is less likely to be a representation of what is happening in the entire country’ | Please refer to Page 17 |
| Data Collection Lines 1-5, Does the standardized question obtain all the information required? Are there pieces of information that should be obtained that are not included in the structured questionnaire? Also, for uneducated patients, how does the team ensure that all the required data is obtained? | All relevant patient characteristics/information determined to have association/possible association with intestinal carriage of ESBL-E among pregnant women from literature reviews and research with similar methodologies were obtained using the structured questionnaire. Notwithstanding, other additional pieces of information could have been added.  Questionnaire was interpreted to un-educated study participants in their local/native languages by a trained personnel | Please refer to Page 6 |
| Page 5: Method should be Methods since several methods were used. | “Method” has been revised to “Methods” We are grateful to the reviewer. | Please refer to Page 6 |
| For all methods used, authors cite their previous authors, especially since these methods did not originate from them. All segments of the materials and methods except for Whole genome sequencing lack any reference | Comment duly addressed. Statements in the methodology section has been duly referenced with in-text citations “1, 11, 12, 13, 14, 15, 16” | Please refer to Pages 6 - 8 |
| Page 9: Result should be Results | “Result” has been revised to “Results” | Please refer to Page 10 |
| Page 9: This statement lacks a full stop, authors should add a full stop:  "About 81% (n = 567) of the participants had a toilet facility at home, 78% (n = 551) had no history of hospitalization in the past year, while 92% (n = 647) had not taken any antibiotics during the same period" | Comment duly addressed. The statement has been revised to include a full-stop | Please refer to Page 10 |
| Page 18: Conclusion, the authors should identify any limitations to the execution of the research as this is necessary and obvious going by the methods used. | Comment duly addressed. Some limitations were observed during the study and have been stated duly in the discussion. | Please refer to Page 18 |
| Reviewer #2 | | |
| Methods Study participants, sampling size and sampling procedure: The authors mentioned......questionnaire was administered to evaluate risk factors for intestinal carriage of ESBL-E (S1_Fig). However, S1_Fig is a flow chart and not a questionnaire. Could you rectify this? | Comment duly addressed. The sentence has been revised to read, ‘A flow chart of how pregnant women were selected and processed is indicated in supplementary Figure 1 (S1 Fig).’ | Please refer to Page 7 |
| Reviewer #3 |  |  |
| Introduction: In lines 3 -5 there is a disjoint between the sentences hence what you are trying to state is not clear; kindly rephrase or insert the correct punctuation | For clarity and logical flow, we have rephrase the statement to read, ‘Extended-spectrum beta-lactamases are enzymes that hydrolyse antibiotics with the beta-lactam ring, including penicillins, broad-spectrum cephalosporins, and monobactams except for carbapenems [2,3]. ESBL-E often exhibit multi-drug resistance, affecting second-line antibiotic therapy for common community-acquired infections like urinary tract infections (UTIs) [4,5]. | Please refer to Page 4 |
| I would like to know what informed your sample size and how you came about it | Given that there is no data on the prevalence of faecal carriage of ESBL-Ec in Korle-Bu Teaching Hospital or elsewhere in Ghana, we used a default value of 50% (0.5). The approach used here is consistent with what is provided in the literature. *(Charan J, Biswas T. How to Calculate Sample Size for Different Study Designs in Medical Research? Indian journal of psychological medicine. 2013;35(2):121–6.)* |  |
| Sample collection and laboratory Investigation: Meropenem 20 microgram was written in Line 17 and the same in Line 20. Kindly address | The Meropenem disk concentration has been revised from 20 µg to 10 µg.  We are most grateful to the reviewers. | Please refer to Page 8 |
|  |  |  |
